# Genome sequences of *Knoxdaviesia capensis* and *K. proteae* (Fungi: Ascomycota) from *Protea* trees in South Africa

**DOI:** 10.1186/s40793-016-0139-9

**Published:** 2016-02-29

**Authors:** Janneke Aylward, Emma T. Steenkamp, Léanne L. Dreyer, Francois Roets, Brenda D. Wingfield, Michael J. Wingfield

**Affiliations:** Department of Botany and Zoology, Stellenbosch University, Private Bag X1, Matieland, 7602 South Africa; Department of Microbiology and Plant Pathology, University of Pretoria, Pretoria, 0002 South Africa; Department of Conservation Ecology and Entomology, Stellenbosch University, Private Bag X1, Matieland, 7602 South Africa; Department of Genetics, University of Pretoria, Pretoria, 0002 South Africa

**Keywords:** *Knoxdaviesia*, *Gondwanamycetaceae*, Microascales, Ophiostomatoid fungi, *Protea*

## Abstract

Two closely related ophiostomatoid fungi, *Knoxdaviesia capensis* and *K. proteae*, inhabit the fruiting structures of certain *Protea* species indigenous to southern Africa. Although *K. capensis* occurs in several *Protea* hosts, *K. proteae* is confined to *P. repens*. In this study, the genomes of *K. capensis* CBS139037 and *K. proteae* CBS140089 are determined. The genome of *K. capensis* consists of 35,537,816 bp assembled into 29 scaffolds and 7940 predicted protein-coding genes of which 6192 (77.98 %) could be functionally classified. *K. proteae* has a similar genome size of 35,489,142 bp that is comprised of 133 scaffolds. A total of 8173 protein-coding genes were predicted for *K. proteae* and 6093 (74.55 %) of these have functional annotations. The GC-content of both genomes is 52.8 %.

**Electronic supplementary material:**

The online version of this article (doi:10.1186/s40793-016-0139-9) contains supplementary material, which is available to authorized users.

## Introduction

Two lineages of the polyphyletic assemblage known as ophiostomatoid fungi [1] are associated with the fruiting structures (infructescences) of serotinous *Protea* L. plants [2]. *Protea* species are a key component of the fynbos vegetation in the Core Cape Subregion (CCR) of South Africa [3] and the genus is predominantly encountered in South Africa [4, 5]. The *Protea*-associated ophiostomatoid fungi are, therefore, believed to be endemic to this region, similar to their hosts. This association of ophiostomatoid fungi with a keystone plant genus in a biodiversity hotspot is intriguing [6], as many ophiostomatoid fungi are notorious pathogens of trees [7–10], yet the *Protea* ophiostomatoid species are not associated with disease symptoms [11].

Ophiostomatoid fungi are characterized by the flask-shaped morphology of their sexual fruiting structures and their association with arthropods [1, 12]. The *Protea*-associated members of this assemblage are primarily dispersed by mites that come into contact with fungal spores in the *Protea* infructescences [13, 14]. These mites have limited dispersal ability, but use beetles and possibly larger vertebrates (such as birds) as vehicles for long-distance dispersal [15, 16].

The three *Knoxdaviesia* M.J. Wingf., P.S. van Wyk & Marasas species associated with *Protea* have intriguing host ranges. *K. capensis* M.J. Wingf. & P.S. van Wyk occurs in at least eight different *Protea* hosts, whereas *K. proteae* M.J. Wingf., P.S. van Wyk & Marasas and *K. wingfieldii* (Roets & Dreyer) Z.W. de Beer & M.J. Wingf. are confined to single host species, respectively *P. repens* L. and *P. caffra* Meisn.[17–20]. An investigation of the population biology of *K. proteae*, revealed that this fungus has a high level of intra-specific genetic diversity and that it is extensively dispersed within the CCR of South Africa [16, 21]. However, other than host range and dispersal mechanisms, little is known about the biology and ecology of *Knoxdaviesia* in general [11]. Here we present the description of the first drafts of the genome sequences of the two CCR species, *K. capensis* and *K. proteae*, as well as their respective annotations.

## Organism information

### Classification and features

**Fig. 1:**
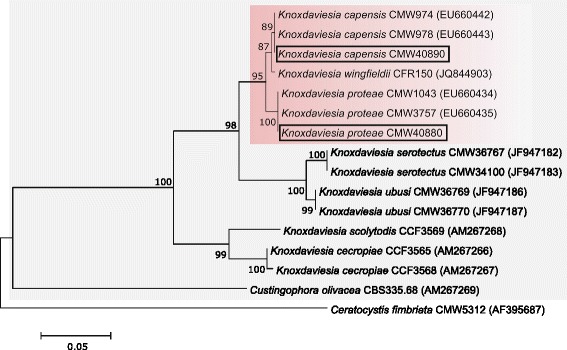
Maximum Likelihood tree illustrating the phylogenetic position of *K. capensis* and *K. proteae* in the *Gondwanamycetaceae* (*grey block*). The *Protea*-associated species are shaded red and the two isolates for which genome sequences were determined are indicated with a box. The sequences of the Internal Transcribed Spacer (ITS) region (available from GenBank®, accession numbers in brackets following isolate numbers) were aligned in MAFFT 7 [55]. The phylogeny was calculated in MEGA6 [56] using the Tamura-Nei substitution model [57], 1000 bootstrap replicates and *Ceratocystis fimbriata* (*Ceratocystidaceae*) as an outgroup

The one lineage of *Protea*-associated ophiostomatoid fungi resides in the *Ophiostomataceae* (Ophiostomatales, Ascomycota), while the second resides in the *Gondwanamycetaceae* (Microascales, Ascomycota) [11, 22]. The latter group includes three closely related *Protea*-associated species in the genus *Knoxdaviesia* (Fig. 1). This genus was initially described to accommodate the asexual state of the first species in the genus, *K. proteae* [23]. Under the dual nomenclature system of fungi, the sexual state of this fungus was described in the same paper as *Ceratocystiopsis proteae* M.J. Wingf., P.S. van Wyk & Marasas [23]. A new genus, *Gondwanamyces* G.J. Marais & M.J. Wingf., was later described to accommodate the sexual state of this species and that of another species, *Ophiostoma**capense* M.J. Wingf. & P.S. van Wyk [24]. The asexual states of both remained to be treated as species of *Knoxdaviesia*. Since the abolishment of the dual nomenclature system of fungi, the oldest genus name takes preference, irrespective of morph [25, 26]. The name *Knoxdaviesia*, therefore, has priority and all species previously treated in *Gondwanamyces* were transferred to *Knoxdaviesia* [27].

**Fig. 2:**
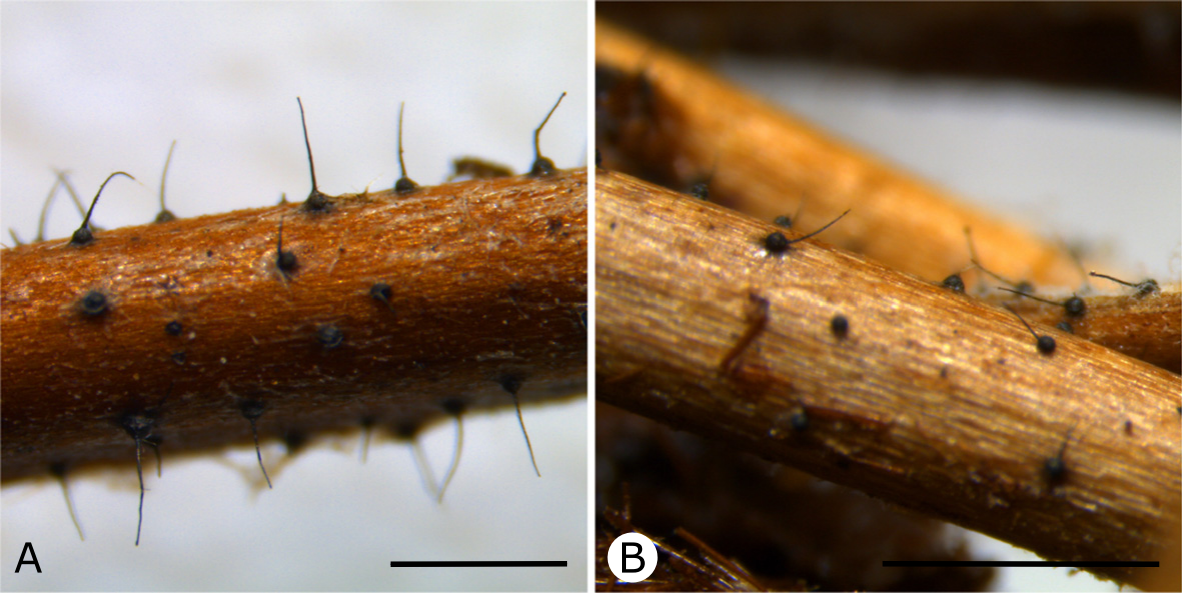
Sexual sporing structures of the two *Knoxdaviesia* species sequenced in this study. *K. capensis* (**a**) and *K. proteae* (**b**) were sampled from *Protea longifolia* and *P. repens* flowers, respectively. Scale bars = 1 mm

In a study determining the genome sequence of any fungus, it is advisable to use a living isolate connected to the type specimen. However, the ex-type isolate of *K. proteae* (CMW738 = CBS486.88) is more than 20 years old and does not display the characteristic morphological features of the fungus in culture anymore. No living ex-type isolate exists for *K. capensis*. We thus collected fresh isolates of both species for this study in order to eliminate possible mutations or degradation that may have occurred though continual artificial propagation in culture media. The new isolates (Figs. 1 & 2) were collected from the same localities and hosts as the holotype specimens: *K. capensis* (CMW40890 = CBS139037) from the infructescences of *P. longifolia* Andrews in Hermanus, and *K. proteae* (CMW40880 = CBS140089) from *P. repens* infructescences in Stellenbosch, both locations in the Western Cape Province of South Africa. General features of these isolates are outlined in Table 1.

**Table 1:** Classification and general features of *K. capensis* and *K. proteae* [29]

MIGS ID	Property	*K. capensis* Term	*K. proteae* Term	Evidence code^a^
	Classification	Domain Fungi	Domain Fungi	TAS [19, 23]
		Phylum Ascomycota	Phylum Ascomycota	TAS [19, 23]
		Class Sordariomycetes	Class Sordariomycetes	TAS [19, 23]
		Order Microascales	Order Microascales	TAS [2]
		Family *Gondwanamycetaceae*	Family *Gondwanamycetaceae*	TAS [22]
		Genus *Knoxdaviesia*	Genus *Knoxdaviesia*	TAS [27]
		Species *K. capensis*	Species *K. proteae*	TAS [27]
		Strain: CMW40890 = CBS139037	Strain: CMW40880 = CBS140089	
	Cell shape	septate, smooth-walled hyphae	septate, smooth-walled hyphae	TAS [19, 23]
	Motility	Non-motile	Non-motile	NAS
	Sporulation	Unsheathed allantoid ascospores	Falcate ascospores	TAS [19, 23]
	Temperature range	15–30 °C	15–30 °C	TAS [19, 23]
	Optimum temperature	25 °C	25 °C	TAS [19, 23]
	pH range; Optimum	Unknown	Unknown	
	Carbon source	Unknown	Unknown	
MIGS-6	Habitat	Seed cones (infructescences) of *Protea* spp.	Seed cones (infructescences) of *Protea repens* L.	TAS [19, 23]
MIGS-6.3	Salinity	Unknown	Unknown	
MIGS-22	Oxygen requirement	Aerobic; requirement/tolerance unknown	Aerobic; requirement/tolerance unknown	
MIGS-15	Biotic relationship	Plant-associated	Plant-associated	TAS [24]
MIGS-14	Pathogenicity	None known	None known	
MIGS-4	Geographic location	Hermanus, South Africa	Stellenbosch, South Africa	
MIGS-5	Sample collection	February 2014	January 2014	
MIGS-4.1	Latitude	-34.4093	-33.9430	
MIGS-4.2	Longitude	19.2150	18.8802	
MIGS-4.4	Altitude	20 m	140 m	

^a^Evidence codes - *IDA* inferred from direct assay, *TAS* traceable author statement (i.e., a direct report exists in the literature), *NAS* non-traceable author statement (i.e., not directly observed for the living, isolated sample, but based on a generally accepted property for the species, or anecdotal evidence). These evidence codes are from http://www.geneontology.org/GO.evidence.shtml of the Gene Ontology project [58]

## Genome sequencing information

### Genome project history

Considering the lack of ecological information on the genus *Knoxdaviesia* and the close relationship these Microascalean fungi have to important plant pathogens, two *Protea*-associated *Knoxdaviesia* species, believed to be native to the CCR in South Africa, were selected for genome sequencing. Both species were sequenced at Fasteris in Switzerland. The genome projects are listed in the Genomes OnLine Database [28] and the whole genome shotgun (WGS) project has been deposited at DDBJ/EMBL/GenBank (Table 2). Table 2 presents the project information and its association with the minimum information about a genome sequence version 2.0 compliance [29]. The full MIGS records for *K. capensis* and *K. proteae* are available in Additional file 1: Table S1 and Additional file 2: Table S2, respectively.

**Table 2:** Project information

MIGS ID	Property	*K. capensis* Term	*K. proteae* Term
MIGS 31	Finishing quality	High quality draft	High quality draft
MIGS-28	Libraries used	2x paired-end (PE) (350 and 550 bp) and 1x mate-pair (MP) (3 kbp)	2x paired-end (PE) (350 and 550 bp) and 1x mate-pair (MP) (3 kbp)
MIGS 29	Sequencing platforms	Illumina Hiseq 2500	Illumina Hiseq 2500
MIGS 31.2	Fold coverage	PE library 1: 91.6 x	PE library 1: 142 x
		PE library 2: 80 x	PE library 2: 79.3 x
		MP library: 17 x	MP library: 50.2 x
MIGS 30	Assemblers	ABySS 1.5.2; SSPACE 3.0	ABySS 1.5.2; SSPACE 3.0
MIGS 32	Gene calling method	MAKER 2.31.8	MAKER 2.31.8
	Genbank ID	LNGK00000000	LNGL00000000
	GenBank Date of Release	11^th^ January 2016	11^th^ January 2016
	GOLD ID	Gp0093999	Gp0110284
	BIOPROJECT	PRJNA246171	PRJNA275563
MIGS 13	Source Material Identifier	CMW40890/CBS139037	CMW40880/CBS 140089
	Project relevance	Biodiversity, evolution	Biodiversity, evolution

### Growth conditions and genomic DNA preparation

Both *K. capensis* and *K. proteae* were cultured on Malt Extract Agar (MEA; Merck, Wadeville, South Africa) overlaid with sterile cellophane sheets (Product no. Z377597, Sigma-Aldrich, Steinham, Germany). After 10 days of growth at 25 °C, mycelia was scraped from the cellophane and DNA was extracted according to Aylward et al. [30]. Approximately 5 μg DNA from each species was used to prepare the three Illumina libraries (Table 2).

RNA was extracted from the *K. proteae* genome isolate to use as evidence for gene prediction. After growth on MEA at 25 °C for approximately 10 days, total RNA was isolated from the mycelia with the PureLink™ RNA Mini Kit (Ambion, Austin, TX, USA). Quality control was performed on the Agilent 2100 Bioanalyzer (Agilent Technologies, USA) using the RNA 6000 Nano Assay kit (Agilent Technologies, USA). The mRNA component of the total RNA was subsequently extracted with the Dynabeads® mRNA purification kit (Ambion, Austin, TX, USA).

### Genome sequencing and assembly

The genomes of *K. capensis* and *K. proteae* were sequenced with the Illumina HiSeq 2500 platform at Fasteris, Switzerland, using two paired-end and one Nextera mate-pair library (Table 2). More than 60 million paired-end and 8 million mate-pair reads were obtained for each species. These reads were trimmed in CLC Genomics Workbench 6.5 (CLC bio, Aarhus, Denmark) so that the Phred *Q* (quality) score of each base was at least Q20. VelvetOptimiser (Gladman & Seeman, unpublished), a Perl script used as part of the Velvet assembler [31, 32], was initially used to optimize the assembly parameters. Assembly of contigs was performed in ABySS 1.5.2 [33] using the optimal parameters suggested by VelvetOptimiser as a starting point. Several assemblies were computed using kmer-values slightly higher and lower than the kmer-value suggested by VelvetOptimiser. The assembly with the lowest number of contigs was used to build scaffolds in SSPACE 3.0 [34], discarding scaffolds smaller than 1000 bp. Automatic gap closure was performed in GapFiller 1.10 [35]. The average genome coverage of each library was estimated using the Lander-Waterman equation (total sequenced nucleotides/genome size) (Table 2), which yielded a combined average coverage for the three libraries of 188.5x (*K. capensis*) and 271.5x (*K. proteae*).

The *K. capensis* genome consists of 29 scaffolds ranging between 1226 and 5,637,848 bp, whereas the 133 scaffolds of *K. proteae* are sized between 1022 and 2,610,973 bp. A search for the 1438 fungal universal single-copy ortholog genes with BUSCO 1.1b1 [36] identified 1355 complete and 67 partial genes in *K. capensis* and 1366 complete and 57 partial genes in *K. proteae*. The two genomes are therefore estimated to be >98 % complete.

The extracted mRNA of *K. proteae* was sequenced using an Ion PI™ Chip on the Ion Proton™ System (Life Technologies, Carlsbad, CA) at the Central Analytical Facility (CAF), Stellenbosch University, South Africa. The >49 million raw RNA-Seq reads were mapped to the *K. capensis* genome in CLC Genomics Workbench and assembled with Trinity 2.0.6 [37] using the genome-guided option.

### Genome annotation

Genome annotation was performed with the MAKER 2.31.8 pipeline [38, 39], using custom repeat libraries for each species constructed with RepeatScout 1.0.5 [40] and two *de novo* gene predictors, SNAP 2006-07-28 [41] and AUGUSTUS 3.0.3 [42]. The assembled *K. proteae* RNA-Seq and predicted protein and/or transcript sequences from 22 sequenced Sordariomycete species (Additional file 3: Table S3), including two Microascalean fungi, were provided as additional evidence. AUGUSTUS was trained with the assembled *K. proteae* RNA-Seq data and subsequently MAKER was used to annotate the largest scaffold of the *K. capensis* and the largest scaffold of the *K. proteae* assembly, independently. After manually curating all the gene predictions on these scaffolds with Apollo 1.11.8 [43], SNAP was trained with the curated gene predictions of each scaffold and the scaffolds were re-annotated. SNAP was re-trained for each species individually and subsequently both genomes were annotated. EuKaryotic Orthologous Group (KOG) classifications were assigned to the predicted proteins through the WebMGA [44] portal that performs reverse-position-specific BLAST [45] searches on the KOG database [46]. Additional functional annotations were predicted with InterProScan 5.13-52.0 [47, 48], SignalP 4.1 [49] and TMHMM 2.0 [50].

## Genome properties

*K. capensis* and *K. proteae* have similar genome sizes at 35.54 and 35.49 Mbp, respectively. It was possible to assemble the *K. capensis* genome into 29 scaffolds larger than 1000 bp, whereas the number of scaffolds above this threshold achieved for *K. proteae* was 133. Both genomes had a GC content of 52.8 %.

A total of 7940 protein-coding genes were predicted for *K. capensis* and 8174 for *K. proteae*. Additionally 137 and 116 tRNA and 30 and 27 rRNA genes were predicted for each species, respectively. More than 74 % of the protein-coding genes of each species could be assigned to a putative function via the KOG and Pfam databases. The content of the two genomes are summarized in Tables 3 and 4.

**Table 3:** Genome statistics

Species	*K. capensis*		*K. proteae*	
Attribute	Value	% of Total^a^	Value	% of Total^a^
Genome size (bp)	35,537,816	100.00	35,489,142	100.00
DNA coding (bp)	12,640,368	35.57	12,542,580	35.34
DNA G + C (bp)	18,774,628	52.83	18,745,365	52.82
DNA scaffolds	29		133	
Total genes	8107	100.00	8316	100.00
Protein coding genes	7940	97.94	8173	98.28
RNA genes^b^	167	2.06	143	1.72
Pseudo genes	unknown		unknown	
Genes in internal clusters	unknown		unknown	
Genes with function prediction	6192	77.98	6093	74.55
Genes assigned to KOGs	6059	76.31	6015	73.60
Genes with Pfam domains	5455	68.70	5335	65.28
Genes with signal peptides	354	4.46	335	4.10
Genes with transmembrane helices	1510	19.02	1527	18.68
CRISPR repeats	N/A		N/A	

^a^The total is based on either the size of the genome in base pairs or the total number of protein-coding genes in the annotated genome

^b^Based on tRNA and rRNA genes only

**Table 4:** Number of genes associated with the 25 general KOG functional categories

Species	*K. capensis*		*K. proteae*		
Code	Value	% of total^a^	Value	% of total^a^	Description
J	359	4.52	371	4.54	Translation, ribosomal structure and biogenesis
A	280	3.53	273	3.34	RNA processing and modification
K	475	5.98	484	5.92	Transcription
L	196	2.47	198	2.42	Replication, recombination and repair
B	109	1.37	99	1.21	Chromatin structure and dynamics
D	209	2.63	227	2.78	Cell cycle control, cell division, chromosome partitioning
Y	34	0.43	32	0.39	Nuclear structure
V	32	0.40	32	0.39	Defence mechanisms
T	505	6.36	586	5.95	Signal transduction mechanisms
M	69	0.87	76	0.93	Cell wall/membrane/envelope biogenesis
N	6	0.08	6	0.07	Cell motility
Z	279	3.51	289	3.54	Cytoskeleton
W	10	0.13	12	0.15	Extracellular structures
U	539	6.79	543	6.64	Intracellular trafficking, secretion, and vesicular transport
O	502	6.32	495	6.06	Post-translational modification, protein turnover, chaperones
C	265	3.34	256	3.13	Energy production and conversion
G	202	2.54	202	2.47	Carbohydrate transport and metabolism
E	227	2.86	228	2.79	Amino acid transport and metabolism
F	76	0.96	74	0.91	Nucleotide transport and metabolism
H	87	1.10	85	1.04	Coenzyme transport and metabolism
I	234	2.95	234	2.86	Lipid transport and metabolism
P	144	1.81	151	1.85	Inorganic ion transport and metabolism
Q	139	1.75	137	1.68	Secondary metabolites biosynthesis, transport and catabolism
R	735	9.26	694	8.49	General function prediction only
S	344	4.33	330	4.04	Function unknown
X	2	0.03	1	0.01	Multiple functions
-	1881	23.69	2159	26.41	Not in KOGs

^a^The total is based on the total number of protein coding genes in the genome

## Conclusions

At least six Microascalean fungi currently have publically accessible genomes [51–54]. *K. capensis* and *K. proteae*, however, represent the first sequenced genomes from the Microascalean family *Gondwanamycetaceae*. The genomes of these two species will not only enable further understanding of the unique ecology of *Protea*-inhabiting fungi, but will also be valuable in taxonomic and evolutionary studies.

## Additional files

Additional file 1:
**Table S1.** Associated MIGS record for *K. capensis*. (DOC 75 kb) Additional file 2:
**Table S2.** Associated MIGS record for *K. proteae*. (DOC 73 kb) Additional file 3:
**Table S3.** Sequenced Sordariomycete fungi used as evidence for genome annotations. (XLSX 12 kb)

## Abbreviations

CCR: core cape subregion
MEA: malt extract agar
KOG: EuKaryotic Orthologous Groups of proteins
