# Supplementary material for: Genome sequences of Knoxdaviesia capensis and K. proteae (Fungi: Ascomycota) from Protea trees in South Africa
Source: Stand Genomic Sci. 2016 Feb 29;11:22. doi: 10.1186/s40793-016-0139-9 (PMC4772463; doi:10.1186/s40793-016-0139-9)
Supplement: Additional file 1: — Table S1. Associated MIGS record for K. capensis. (DOC 75 kb) [file 40793_2016_139_MOESM1_ESM.doc]

# Associated MIGS Record

**Table S1. Associated MIGS record for** [***K. capensis***](http://www.ncbi.nlm.nih.gov/Taxonomy/Browser/wwwtax.cgi?lvl=0&id=114771)

| **MIGS-ID** | field name | description |
| --- | --- | --- |
| **MIGS-1** | Submit to INSDC/Trace archives |  |
| **1.1** | PID |  |
| **1.2** | Trace Archive |  |
| **MIGS-2** | MIGS CHECK LIST TYPE | Eukaryote |
| **MIGS-3** | Project Name | [*K. capensis*](http://www.ncbi.nlm.nih.gov/Taxonomy/Browser/wwwtax.cgi?lvl=0&id=114771) H9 genome sequencing |
| **MIGS-4** | Geographic Location | South Africa: Western Cape Province: Hermanus |
| **4.1** | Latitude | -34.4093 |
| **4.2** | Longitude | 19.2150 |
| **4.3** | Depth |  |
| **4.4** | Altitude |  |
| **MIGS-5** | Time of Sample collection | 2014-02-14 |
| **MIGS-6** | Habitat (EnvO) | plant-associated |
| **6.1** | temperature |  |
| **6.2** | pH |  |
| **6.3** | salinity |  |
| **6.4** | chlorophyll |  |
| **6.5** | conductivity |  |
|
| **6.6** | light intensity |  |
| **6.7** | dissolved organic carbon (DOC) |  |
| **6.8** | current |  |
| **6.9** | atmospheric data |  |
| **6.10** | density |  |
| **6.11** | alkalinity |  |
| **6.12** | dissolved oxygen |  |
| **6.13** | particulate organic carbon (POC) |  |
| **6.14** | phosphate |  |
| **6.15** | nitrate |  |
| **6.16** | sulfates |  |
| **6.17** | sulfides |  |
| **6.18** | primary production |  |
| **MIGS-7** | Subspecific genetic lineage |  |
| **MIGS-8** | Ploidy | Haploid |
| **MIGS-9** | Number of replicons | unknown |
| **MIGS-10** | Extrachromosomal elements |  |
| **MIGS-11** | Estimated Size | 35,000,000 |
| **MIGS-12** | Reference for biomaterial or Genome report | (this manuscript) |
| **MIGS-13** | Source material identifiers | [CBS 139037](http://doi.org/10.1601/strainfinder?urlappend=%3Fid%3DCBS+139037); [CMW 40890](http://doi.org/10.1601/strainfinder?urlappend=%3Fid%3DCMW+40890) |
| **MIGS-14** | Known Pathogenicity | Non-pathogen |
|
| **MIGS-15** | Biotic Relationship | Symbiont |
| **MIGS-16** | Specific Host | [*Protea longifolia*](http://www.theplantlist.org/tpl1.1/record/tro-26500475) (TaxID 73576) |
| **MIGS-17** | Host specificity or range (taxid) | [*Protea*](http://www.ncbi.nlm.nih.gov/Taxonomy/Browser/wwwtax.cgi?lvl=0&id=4331)spp. (TaxID 4331) |
| **MIGS-18** | Health status of Host | Asymptomatic |
| **MIGS-19** | Trophic Level | Heterotroph |
| **MIGS-20** | Propagation | Sexual and asexual |
| **MIGS-22** | Relationship to Oxygen | Aerobic |
| **MIGS-23** | Isolation and Growth conditions | DOI: 10.1007/s11557-013-0951-1 |
| **MIGS-27** | Nucleic acid preparation | PMCID: PMC334490 |
| **MIGS-28** | Library construction | Paired-end |
| **28.1** | Library size | 350, 550, 3 000 |
| **28.2** | Number of reads |  |
| **28.3** | vector |  |
| **MIGS-29** | Sequencing method | Illumina HiSeq 2500 |
| **MIGS-30** | Assembly |  |
| **30.1** | Assembly method | ABYSS 1.5.2, SSPACE 3.0 |
| **30.2** | estimated error rate | 1 in 100 bp |
| **30.3** | method of calculation | PHRED |
| **MIGS-31** | Finishing strategy |  |
| **31.1** | Status | High-quality draft |
| **31.2** | coverage |  |
| **31.3** | contigs | 29 |
| **MIGS-32** | Relevant SOPs |  |
| **MIGS-33** | Relevant e-resources |  |
